# Quality of Life Among Children With Type 1 Diabetes Mellitus in Alahsa: A Cross-Sectional Study

**DOI:** 10.7759/cureus.40766

**Published:** 2023-06-21

**Authors:** Jumanah A Alhaddad, Nariman A Alshakes, Majdi N Aljasim

**Affiliations:** 1 Joint Residency Program Preventive Medicine, Al-Ahsa Health Cluster, Ministry of Health, Alahsa, SAU; 2 Model of Care, Rural Health Network, First Eastern Cluster, Ministry of Health, Alahsa, SAU; 3 Research and Public Health Unit, Department of Preventive Medicine, Al-Ahsa Health Cluster, Ministry of Health, Alahsa, SAU

**Keywords:** adolescent, child health, saudi arabia, quality of life, diabetes mellitus

## Abstract

Background: Diabetes mellitus (DM) is the most common endocrine disease in children, and its annual prevalence is increasing worldwide. Health-related quality of life (HRQoL) is a crucial indicator of chronic illnesses such as DM. This study aimed to assess the HRQoL and determine the associated factors among children and adolescents with type 1 DM in Alahsa region in 2022.

Methods: An analytical cross-sectional study was conducted in the DiabeterSA center using the Arabic version of the Pediatric Quality of Life Inventory (PedsQl 3.2). All patients aged 5-16 years and diagnosed with type 1 DM were included in the study. Face-to-face interviews were conducted during the patients’ routine visits to the outpatient clinic from September 2022 to January 2023.

Result: A total of 254 children aged 5-16 years (mean age: 10.87 ± 2.89 years) were recruited. The mean HRQoL total score reported by the children and adolescents was 72.61 ± 15.36. Older age, higher family socioeconomic status, excellent school performance, and higher parental education level, particularly in mothers, were significantly associated with higher total HRQoL scores. In the regression analysis, male sex (β = -0.157,P = 0.018), older age (β = 0.246, P <0.001), and excellent school performance (β = -0.290, P < 0.001) were identified as independent predictors of the HRQoL total score. Nearly 19% of the participants achieved glycemic control.

Conclusion: The quality of life of children and adolescents with type 1 DM in Alahsa region was relatively good. Increased age, good family economic status, and higher parent education levels positively influenced the participants’ quality of life. Therefore, regular evaluation of HRQoL is recommended for children and adolescents with type 1 DM to identify problems and initiate an appropriate intervention for improving child health and the health care system.

## Introduction

Diabetes mellitus (DM) is the most frequent endocrine disease among children [1]. Diabetes mellitus is a metabolic disease characterized by chronic hyperglycemia [2]. According to the American Diabetes Association (AHA) classification, diabetes is categorized into type 1 DM (T1DM), which can be attributed to β-cell destruction and insufficient insulin; type 2 DM, which occurs due to insulin resistance; gestational diabetes mellitus, and diabetes attributable to other specific causes [3]. Globally, the prevalence of DM is increasing by 3%-4% every year [4].

Saudi Arabia (SA) is the largest country in the Middle East, with an estimated population of 35 million, of which approximately 30% are younger than 14 years of age [5]. Saudi Arabia shows the most significant prevalence of DM among children in the Middle East and North Africa [4]. The approximate prevalence of T1DM among children and adolescents in SA is 109.5 per 100,000, and the highest and lowest prevalences have been reported in the central and eastern regions (126 per 100,000 and 48 per 100,000, respectively) [1,2,6]. Type 1 DM requires treatment with specific drugs and follow-up for daily multiple insulin injections, a diet with carbohydrate calculation, maintenance of physical activity, and continuous glucose monitoring (CGM) to achieve control of glycemic blood levels and prevent macrovascular and microvascular complications [2,3]. Health-related quality of life (HRQoL) is an essential indicator in medical conditions, especially chronic diseases such as DM [7], and the achievement of optimal HRQoL is a tremendously challenging goal for patients with diabetes and their families [8].

Many studies have reported poor quality of life (QoL) among children with T1DM in comparison with their healthy peers [9,10]. Moreover, systematic reviews and meta-analyses have suggested that greater QoL is associated with better glycemic control in the pediatric population with T1DM [2,11]. However, studies addressing HRQoL and its relationship to glycemic control among children and adolescents with T1DM in Gulf countries are limited. In Kuwait, a case-control study compared QoL among children with T1DM and matched healthy children, and the findings showed poorer QoL among patients with diabetes [12]. Özyazıcıoğlu et al. further pointed out that the worst QoL was associated with high glycosylated hemoglobin (HBA1c) levels. In another recent study in Jeddah, SA [13] showed that girls with T1DM have poorer QoL than boys, and a significant correlation between glycemic control and the QoL score for the communication subgroup was identified [13].

Although the eastern region in SA has the lowest prevalence of T1DM, the prevalence is still higher than that in the Gulf region and globally, and to our knowledge, no published study has addressed HRQoL among children in the eastern province or children and adolescents in Alahsa region. Therefore, this study assessed HRQoL among children and adolescents with T1DM in Alahsa region, determined potential factors influencing glycemic control and QoL, and evaluated the correlation between QoL and glycemic control.

## Materials and methods

This cross-sectional analytical study was conducted using a validated Arabic version survey [4]. Ethical approval was obtained from Alahsa Health Cluster Institutional Review Board (IRB Log No.: 31-EP-2022), and the study was performed according to the Helsinki Declaration. In addition, each patient was coded to maintain confidentiality.

This study was conducted in Alahsa, which is located in the Eastern Province of Saudi Arabia, approximately 320 km east of Riyadh, the capital city of SA, and approximately 97 km inland from the Arabian Gulf. Alahsa consists of four major cities: Hofuf, Al-Mobarraz, Al-Oyoun, and Al-Oman. Only one governmental referral hospital, a pediatric diabetes center (DiabeterSA), has recently followed the pediatric age group with diabetes in this region. DiabeterSA covers the follow-up of all referral T1DM cases from all governmental hospitals in Alahsa. To the best of our knowledge, the estimated number of registered patients is 750 till January 2023. All children and adolescents aged 5-16 years and diagnosed with T1DM were eligible to participate in this study, and all patients with diabetes who underwent follow-up assessments at the outpatient clinic at DiabeterSA were also included. However, patients with a recent diagnosis of T1DM (less than six months) or any hematological disease or other comorbidities that could affect the QoL, such as an autoimmune or neurological disease, were excluded.

Demographic data, such as the age and sex of the children, were collected. Socioeconomic status was determined on the basis of the family's economic status, the child's school performance, and the parents’ educational levels. Disease-related factors included the duration of illness, type of insulin, type of glucometer used in the last month, number of emergency department visits, and number of diabetic ketoacidoses in the last year. Glycemic control, indicated by the previous four HbA1c readings, was retrospectively collected from the medical records.

Health-related QoL was assessed by a Diabetes Quality of Life Questionnaire (PedsQoL 3.2 Arabic version), a validated tool for evaluating the QoL among children with T1DM, and the Cronbach’s alpha of the tool was 0.894 [5]. This tool is designed to consider the QoL in the past month (30 days). The questionnaire was acquired from eprovide.mapi-trust.org with permission (Special Terms No.: 2207614, June 30, 2022). This is a platform launched in March 2016 by Mapi Research Trust to facilitate access to information for all stakeholders in the field of patient-centered outcomes, particularly for clinical outcome assessments. Three forms of the questionnaire were used; all had similar questions but used understandable language for the target age group (5-7 years old, 8-12 years old, or 13-18 years old). The questionnaire contained 33 items across the following five domains, each of which assessed one aspect of QoL: “diabetes symptoms,” “treatment barriers,” “treatment adherence,” “anxiety,” and “communication.” The score for each question ranged from 0 to 4, and the final score ranged from 0 to 100, with higher scores indicating a better QoL.

The purpose of this study was explained to the parents, and verbal consent was obtained from them and the children. Completion of the survey was estimated to require 5-10 min. Data were collected by the primary investigator using consecutive sampling techniques in face-to-face interviews with the children and their parents during routine visits to the DiabeterSA outpatient clinics from September 2022 to January 2023.

The multicollinearity Pearson's correlation coefficient was used to elaborate on the relationships between different independent variables and the QoL score. Bivariable and multivariable linear regressions were performed to explore the truly significant factors influencing QoL in children and adolescents. Significance for all tests was determined by P-values < 0.05.

The calculated sample size was based on the latest reported prevalence of T1DM among children and adolescents in SA (109 per 100,000) [6] using the Roasoft calculator with a 5% margin of error and 95% confidence interval, and the estimated calculated sample size was 246 participants. Data analysis was conducted using the Statistical Package for the Social Sciences (SPSS), version 25 (IBM Corp., Armonk, New York). Continuous variables were shown as mean ± standard deviation (SD) and percentages as frequency data. In addition, one-way analysis of variance (ANOVA) with posthoc analysis and independent t-test were performed to compare the normally distributed variables.

## Results

A total of 254 children and adolescents with T1DM participated in the study. Of these, 58% were female. The response rate was > 95%. The mean age of the participants was 10.87 ± 2.89 years (10.51 ± 2.95 years for males and 11.12 ± 2.82 years for females). The majority of patients had DM for one to five years (66.54%), and most of the participants were using a sensor or CGM device as the glucometer (77.95%), while the rest were self-monitoring blood glucose (SMBG). Assessments of treatment regimens showed that 93% of the study population was on multiple drug injections (MDI), and only 7% were receiving insulin pump therapy. Glycemic control was defined as a mean HbA1c of less than 7.5% according to the American Diabetic Association (6), and only 19% of participants had optimal glycemic control (mean HbA1c < 7.5). Two-thirds (67%) of the patients had fair family economic status, 27% had good financial status, and 6% had poor economic status. Approximately half of the children and adolescents had parents with university degrees (49% of fathers and 42% of mothers), followed by high school (28% of fathers and 36% of mothers). The participants' demographic characteristics are summarized in Table 1.

**Table 1:** Demographic data of 254 children and adolescents with type 1 diabetes mellitus included in the study *CGM: Continuous glucose monitoring; **SMBG: Self-monitoring blood glucose; ^ⱡ^Young child to attending school (<6 years). SD: Standard deviation; HbA1c: Glycosylated hemoglobin.

Variables		Number	%
Sex	Male	106	41.6
	Female	148	58
Age	5-7 years	39	15.3
	8-12 years	128	50.2
	13-16 years	87	34.1
Economic status	Good	68	26.7
	Fair	171	67.1
	Poor	15	5.9
School performance	Excellent	160	62.7
	Very good	67	26.4
	Good	10	3.9
	No school^ⱡ^	17	6.69
Father’s education	University	122	47.8
	High	69	27.1
	Intermediate	31	12.2
	Primary	19	7.5
	Lower	8	3.1
Mother’s education	University	112	43.9
	High	97	38
	Intermediate	33	12.9
	Primary	6	2.4
	Lower	3	1.2
Duration	Less than a year	2	0.8
	1-5 years	169	66.54
	More than 5 years	83	32.4
Type of insulin	Multiple drug injections	237	92.9
	Pump	17	6.7
Glucometer	Sensor (CGM)*	198	77.95
	SMBG**	56	22
Emergency room visit	None	202	79.2
	1-2 times	34	13.3
	More than 2	13	5.1
Diabetic ketoacidosis	None	215	84.3
	1-2 times	29	11.4
	More than 2	10	3.9
Glycemic control	<7.5	46	18
	>7.5	208	81.3
Variable		Mean	SD
HbA1c		9.04	1.57
Age		10.88	9.04
Diabetes symptoms		68.40	14.87
Treatment barriers		71.32	21.77
Treatment adherence		74	22.31
Worry		75.33	29.61
Communication		74.04	29.80
Total		72.62	15.37

The mean total QoL score reported by the children and adolescents was 72.61 ± 15.36. Boys had a superior total PedsQl Diabetes Module score than girls (mean ± SD: 74.48 ± 14.63 vs. 71.28 ± 15.78). However, the two sexes showed no significant difference in the total HRQoL score (P = 0.55; 95% confidence interval (CI): -0.63 to 7.03). Girls reported significantly worse scores in the diabetes symptoms domain than boys (66.52 ± 15.96 vs. 71.01 ± 13.27; P = 0.017; 95% CI: 0.79 to 8.18). Adolescents (aged 13-16 years) had better scores for treatment barriers than younger children aged five to seven years (77.12 ± 20.03 vs. 65.38 ± 21.44; P = 0.01; 95% CI: -21.46 to -2.01). Moreover, in comparison with children aged five to seven years, older patients aged 8-16 years reported higher scores in the treatment adherence domain (59.87 ± 24.50 vs. 82 ± 19.25; P < 0.001; 95% CI: 12.517 to 31.7436), while adolescents scored higher than children aged five to seven years in a treatment adherence domain (82 ± 19.25 vs. 72.87 ± 21.30; P = 0.006; 95% CI: 2.199 to 16.062). For the communication domain, patients aged five to seven years scored lower than those aged 13-16 years (60.89 ± 35.95 vs. 81.60 ± 22.17; P = 0.01; 95% CI: -33.94 to -7.48). Children aged five to seven years had a lower overall score than adolescents (67.96 ± 15 vs. 72.61 ± 15.36; P < 0.01; 95% CI: 0.58 to 1.96). On the other hand, children from the younger age group (five to seven years) had better glycemic control than adolescents (8.19 ± 1.33 vs. 9.47 ± 1.56, P < 0.001; 95% CI: -1.99 to -0.55). No significant difference was observed between females and males in relation to glycemic control (P = 0.17; 95% CI: -0.54 to -0.25). Among children and adolescents with T1DM, sensor and CGM device users recorded a more substantial improvement in glycemic control than SMBG users (8.9 for CGM vs. 9.5 for SMBG; P = 0.008). However, the QoL scores showed no difference between the two groups. Although the QoL and glycemic control of patients with insulin pumps were higher than those of patients with MDI, no significant difference was observed between the groups (Table 2).

**Table 2:** The scores of the Pediatric Quality of Life Inventory 3.2 Diabetes Module subscales among children and adolescents with type 1 diabetes mellitus *The mean difference is significant at the 0.05 level; ^ⱡ^Young child to attending school (<6 years). HbA1c: Glycosylated hemoglobin; SMBG: Self-monitoring blood glucose; MDI: Multiple drug injections; ER: Emergency room; DKA: Diabetic ketoacidosis; ANOVA: Analysis of variance; ref: Best score set as a reference in the posthoc ANOVA test.

Variables		Diabetes symptoms (P-value)	Treatment barriers (P-value)	Treatment adherence (P-value)	Worry (P-value)	Communication (P-value)	Total (P-value)	Mean HbA1c (P-value)
Sex	Male	71.02 (ref)	73.07 (ref)	75.82 (ref)	78.46 (ref)	74.05 (ref)	74.48 (ref)	8.96 (ref)
	Female	*66.53 (0.02)	70.07 (0.28)	72.70 (0.27)	73.09 (0.15)	74.029 (0.99)	71.28 (0.10)	9.11 (0.46)
Age	5-7 years	71.62 (ref)	*65.38 (0.01)	*59.87 (0.00)	82.05 (0.37)	*60.90 (0.00)	*67.97 (0.01)	8.20 (ref)
	8-12 years	68.10 (0.40)	*69.18 (0.02)	*72.87 (0.01)	74.80 (0.26)	72.90 (0.08)	71.57 (0.07)	*9.01 (0.01)
	13-16 years	67.41 (0.31)	77.13 (ref)	82.00 (ref)	73.08 (ref)	81.61 (ref)	76.25 (ref)	*9.47 (0.00)
Economic status	Good	70.34 (0.48)	74.93 (0.98)	77.12 (ref)	81.00 (ref)	80.33 (ref)	76.74 (ref)	8.95 (0.99)
	Fair	67.85 (0.55)	69.47 (0.50)	72.74 (0.36)	73.73 (0.20)	71.49 (0.10)	*71.06 (0.03)	9.09 (0.90)
	Poor	65.89 (ref)	76.00 (ref)	74.22 (0.89)	67.78 (0.26)	74.58 (0.78)	71.69 (0.48)	8.91 (ref)
School performance	Excellent	69.48 (ref)	76.56 (ref)	78.82 (ref)	76.41 (ref)	79.57 (ref)	76.17 (ref)	8.97 (0.64)
	Very good	67.34 (0.75)	*63.88 (0.00)	*68.87 (0.01)	70.65 (0.54)	69.78 (0.09)	*68.10 (0.00)	9.23 (0.30)
	Good	59.00 (0.13)	*54.00 (0.01)	*58.25 (0.02)	67.50 (0.79)	*48.13 (0.00)	*57.37 (0.00)	9.97 (0.08)
	No school^ⱡ^	67.94 (0.98)	*61.47 (0.02)	*58.14 (0.00)	88.24 (0.39)	*54.04 (0.00)	*65.97 (0.03)	8.49 (ref)
Father's education	University	69.40 (ref)	73.28 (ref)	76.44 (ref)	77.53 (1.00)	74.69 (0.98)	74.27 (ref)	8.80 (ref)
	High	68.21 (0.98)	70.65 (0.93)	73.91 (0.94)	68.72 (0.63)	75.91 (0.99)	71.48 (0.74)	9.05 (0.81)
	Intermediate	64.03 (0.38)	*60.48 (0.03)	*63.82 (0.04)	70.70 (0.85)	70.77 (0.88)	65.96 (0.06)	9.39 (0.31)
	Primary	67.28 (0.98)	70.00 (0.97)	68.38 (0.57)	79.39 (ref)	78.95 (0.19)	72.80 (1.00)	9.55 (0.27)
	Lower	68.13 (1.00)	85.00 (0.56)	85.00 (0.82)	95.83 (0.68)	51.56 (ref)	77.10 (0.99)	10.02 (0.19)
Mother's education	University	69.68 (0.94)	71.56 (0.28)	73.54 (0.43)	77.98 (0.90)	74.94 (ref)	73.54 (0.56)	*8.55 (0.03)
	High	67.03 (0.76)	72.42 (0.33)	77.00 (0.67)	72.25 (0.66)	76.35 (1.00)	73.01 (0.51)	*9.17 (0.00)
	Intermediate	66.11 (0.72)	*60.15 (0.02)	*61.36 (0.04)	70.45 (0.62)	67.42 (0.70)	65.10 (0.06)	10.07 (0.47)
	Primary	74.44 (ref)	89.17 (ref)	89.17 (ref)	88.89 (ref)	73.96 (1.00)	83.13 (ref)	9.58 (0.59)
	Lower	68.80 (0.98)	93.33 (1.00)	93.33 (1.00)	100 (0.98)	31.25 (0.09)	77.36 (0.98)	9.82 (ref)
Duration	Less than a year	60.00 (0.82)	70.00 (0.98)	68.75 (0.82)	*16.67 (0.01)	78.13 (ref)	58.71 (0.39)	8.28 (ref)
	1-5 years	69.53 (0.24)	70.65 (0.76)	71.90 (0.07)	77.37 (ref)	73.08 (0.99)	72.50 (0.94)	8.75 (0.90)
	More than 5 years	66.31 (ref)	72.71 (ref)	78.41 (ref)	72.60 (0.44)	75.90 (0.76)	73.18 (ref)	9.67 (0.40)
Type of insulin	MDI	68.04 (0.15)	70.82 (0.18)	73.44 (0.13)	75.21 (0.81)	73.81 (0.65)	72.27 (0.17)	9.08 (0.16)
	Pump	73.43 (ref)	78.24 (ref)	81.86 (ref)	76.96 (ref)	77.21 (ref)	77.54 (ref)	8.53 (ref)
Glucometer	Sensor	68.89 (ref)	72.20 (ref)	75.02 (ref)	77.27 (ref)	74.43 (ref)	73.56 (ref)	8.91 (ref)
	SMBG	66.69 (0.33)	68.21 (0.23)	70.40 (0.17)	*68.45 (0.05)	72.66 (0.69)	69.28 (0.07)	*9.53 (0.01)
ER visit	None	70.04 (ref)	71.49 (ref)	73.96 (ref)	76.69 (0.12)	73.27 (ref)	73.09 (ref)	8.85 (ref)
	1-2 times	64.01 (0.07)	70.00 (0.93)	76.89 (0.76)	65.93 (0.12)	79.412 (0.51)	71.25 (0.80)	*9.55 (0.04)
	More than 2 times	*57.18 (0.00)	70.77 (0.98)	69.36 (0.83)	69.23 (0.80)	76.44 (0.97)	68.60 (0.48)	*10.69 (0.00)
Diabetic ketoacidosis	None	69.93 (ref)	71.28 (ref)	73.94 (ref)	76.32 (ref)	73.14 (ref)	72.92 (ref)	8.91 (ref)
	1-2 times	64.48 (0.13)	73.79 (0.83)	77.16 (0.75)	70.69 (0.60)	84.27 (0.14)	74.078 (0.92)	9.54 (0.10)
	More than 2 times	*47.00 (0.00)	65.00 (0.65)	66.25 (0.54)	67.50 (0.63)	63.75 (0.59)	61.90 (0.07)	*10.46 (0.01)
Glycemic control	<7.5	72.82 (ref)	72.83 (ref)	73.53 (ref)	78.99 (ref)	75.27 (ref)	74.69 (ref)	6.91 (ref)
	>7.5	*67.43 (0.03)	70.98 (0.60)	74.11 (0.87)	74.52 (0.36)	73.77 (0.76)	72.16 (0.31)	*9.52 (0.00)

Multivariate linear regression indicated that male sex, older age, and school performance were independent influencing factors of the total QoL score (Table 3).

**Table 3:** Results of regression analyses with ß-coefficient and 95% confidence intervals for Pediatric Quality of Life Inventory 3.2 Diabetes Module domains (n = 254) HbA1c: Glycosylated hemoglobin. *The mean difference is significant at the 0.05 level.

Variables		ß-coefficient	95% confidence interval		P-value
			Lower	Upper	
Diabetes symptoms	Sex	-0.15	-8.53	-0.82	0.02*
	Age	-0.03	-3.99	2.59	0.68
	Economic status	-0.05	-5.11	2.50	0.50
	School performance	-0.10	-6.04	0.92	0.15
	Father's education	-0.10	-3.61	0.82	0.22
	Mother’s education	0.06	-1.81	3.83	0.48
	Duration	-0.03	-5.22	3.27	0.65
	Type of insulin	0.10	-2.07	13.13	0.15
	Glucometer	0.00	-4.84	4.77	0.99
	Emergency room visit	-0.02	-4.89	3.98	0.84
	Diabetic ketoacidosis	-0.25	-13.15	-1.87	0.01*
	HbA1c	-0.05	-1.87	0.92	0.50
Treatment barrier	Sex	-0.13	-11.40	-0.10	0.05*
	Age	0.19	1.91	11.55	0.01*
	Economic status	0.02	-4.87	6.29	0.80
	School performance	-0.30	-16.74	-6.54	0.00*
	Father's education	-0.06	-4.44	2.05	0.47
	Mother's education	0.07	-2.33	5.95	0.39
	Duration	0.00	-6.31	6.14	0.98
	Type of insulin	0.06	-6.14	16.14	0.38
	Glucometer	-0.11	-12.82	1.26	0.11
	Emergency room visit	-0.07	-9.09	3.92	0.43
	Diabetic ketoacidosis	0.01	-7.85	8.68	0.92
	HbA1c	0.02	-1.74	2.35	0.77
Treatment adherence	Sex	-0.12	-10.88	0.37	0.07
	Age	0.29	5.62	15.23	0.00*
	Economic status	0.01	-5.29	5.82	0.92
	School performance	-0.25	-15.10	-4.94	0.00*
	Father's education	-0.10	-5.34	1.13	0.20
	Mother's education	0.10	-1.61	6.63	0.23
	Duration	0.04	-4.20	8.20	0.53
	Type of insulin	0.04	-7.27	14.92	0.50
	Glucometer	-0.11	-12.85	1.19	0.10
	Emergency room visit	-0.03	-7.39	5.56	0.78
	Diabetic ketoacidosis	-0.04	-10.01	6.46	0.67
	HbA1c	-0.02	-2.37	1.70	0.74
Worry	Sex	-0.06	-11.83	4.45	0.37
	Age	0.00	-6.76	7.14	0.96
	Economic status	-0.14	-15.92	0.16	0.05*
	School performance	-0.08	-11.63	3.08	0.25
	Father's education	0.03	-3.73	5.63	0.69
	Mother’s education	0.06	-3.71	8.22	0.46
	Duration	0.02	-7.98	9.96	0.83
	Type of insulin	0.00	-16.36	15.76	0.97
	Glucometer	-0.10	-17.22	3.09	0.17
	Emergency room visit	0.03	-7.91	10.83	0.76
	Diabetic ketoacidosis	-0.11	-18.68	5.16	0.26
	HbA1c	-0.02	-3.32	2.58	0.81
Communication	Sex	-0.05	-10.75	4.60	0.43
	Age	0.19	2.50	15.61	0.01*
	Economic status	0.03	-6.10	9.06	0.70
	School performance	-0.20	-17.39	-3.52	0.00*
	Father's education	0.09	-1.76	7.07	0.24
	Mother's education	-0.18	-12.00	-0.74	0.03*
	Duration	-0.02	-9.62	7.30	0.79
	Type of insulin	-0.03	-18.23	12.05	0.69
	Glucometer	-0.04	-12.37	6.78	0.57
	Emergency room visit	-0.18	-17.29	0.39	0.06
	Diabetic ketoacidosis	0.11	-4.44	18.04	0.23
	HbA1c	0.00	-2.86	2.70	0.96
Total	Sex	-0.16	-9.01	-0.84	0.02*
	Age	0.25	0.69	2.24	0.00*
	Economic status	-0.04	-4.87	2.83	0.60
	School performance	-0.29	-11.51	-4.49	0.00*
	Father's education	-0.02	-2.60	1.88	0.75
	Mother’s education	0.02	-2.51	3.19	0.81
	Duration	0.04	-2.67	5.20	0.53
	Type of insulin	0.03	-5.64	9.41	0.62
	Glucometer	-0.11	-8.90	0.65	0.09
	Emergency room visit	-0.08	-6.50	2.45	0.37
	Diabetic ketoacidosis	-0.05	-7.41	3.98	0.55
	HbA1c	-0.10	-1.43	-3.52	0.47
Mean HbA1c	Sex	-0.01	-0.42	0.34	0.83
	Age	0.18	0.11	0.78	0.01*
	Economic status	-0.04	-0.50	0.25	0.50
	School performance	0.10	-0.07	0.64	0.12
	Father's education	0.03	-0.17	0.27	0.64
	Mother’s education	0.28	0.25	0.79	0.00*
	Duration	0.26	0.44	1.23	0.00*
	Type of insulin	-0.11	-1.43	0.05	0.07
	Glucometer	0.03	-0.37	0.57	0.68
	Emergency room visit	0.16	-0.04	0.83	0.07
	Diabetic ketoacidosis	0.02	-0.49	0.63	0.80
	HbA1c				
	Diabetes symptoms	0.01	0.93	-0.02	0.02*
	Treatment barrier	0.20	0.30	-0.01	0.04*
	Treatment adherence	0.05	0.39	0.00	0.01*
	Worry	0.10	0.49	-0.01	0.02*
	Communication	0.12	0.44	-0.01	0.02*
	Total	-0.32	0.39	-0.10	0.04*

The multicollinearity Pearson's correlation coefficient showed no significant correlation between the HbA1c level and the total HRQoL score as well as the scores for all subdomains, except the diabetes symptoms domain (P = 0.02; Table 4).

**Table 4:** Pearson correlation coefficients between mean glycosylated hemoglobin and total score as well as the five domains of health-related quality of life score *Correlation is significant at the 0.05 level (two-tailed). **Correlation is significant at the 0.01 level (two-tailed).

Variables		Age	Score for symptoms	Score for barrier	Score for adherence	Score for worry	Score for communication	Total
Mean HbA1c	Pearson correlation	0.278**	-0.143*	0.02	0.05	-0.06	0.02	-0.02
	Sig. (two-tailed)	0.00	0.02	0.71	0.39	0.35	0.73	0.75

## Discussion

Studies have shown that adolescents with diabetes, particularly females, report lower QoL than males with a similar condition [5,7,8]. Our results showed lower scores of total HRQoL and all subdomains without significant differences between both sexes, except for diabetic symptoms. Moreover, regression analysis showed that the male sex was an independent predictor for the total HRQoL score and the score for diabetic symptoms. Many studies have reported similar results; cross-sectional studies showed that female children and adolescents with T1DM had lower QoL scores than males [9,10]. This could be attributed to cultural and hormonal issues since males had greater access to health services and girls experienced pubertal changes earlier than boys [7].

The challenges faced by children and adolescents with T1DM can vary depending on their age, with younger children struggling with frequent injections or blood glucose monitoring. In contrast, older adolescents may face social stigma or the challenge of independently managing their condition [11]. Our study showed that adolescents had higher total HRQoL scores than younger children, while children (aged five to seven years) had better glycemic control than adolescents. This difference may be due to the closer follow-up from caregivers and parents for children than adolescents. The results presented by Gonder-Frederick et al. support our findings that younger children (aged 8-11 years) with T1DM reported lower QoL scores than older children and adolescents (aged 12-18 years) [11]. Conversely, children had a better HRQoL score than adolescents in Turkey and Addis Ababa [12,13].

The findings of this study suggest that the higher socioeconomic status of children and adolescents with T1DM had a significant positive impact on the total HRQoL score but had a better but not significant impact on glycemic control. A study published in 2017 found that higher family income was associated with better glycemic control in children and adolescents with T1DM. The study also found that higher family socioeconomic status was associated with lower levels of diabetes-related family conflict and better QoL scores [11]. One potential explanation for the association between higher economic status and better diabetes management is access to healthcare resources. Families with higher incomes may have greater access to healthcare services and resources, such as diabetes education, specialized care, and technology, which can help enhance glycemic control and QoL [11,14]. Another possible explanation is the effect of stress and family dynamics on diabetes management. Children and adolescents from lower socioeconomic backgrounds may experience greater stress and family conflicts related to their condition, making it more challenging to effectively manage their diabetes [15]. Education and awareness may also influence the association between socioeconomic status and diabetes management. Families with higher incomes may be more aware of the importance of diabetes management and may have a better understanding of how to manage the condition effectively [15].

School performance, QoL, and glycemic control among children and adolescents showed bidirectional results. Children and adolescents with T1DM who performed well in school tended to have better QoL. They were also more likely to have good self-esteem, better coping skills, and higher levels of social support, all of which can contribute to improved overall well-being [16]. Good school performance was associated with better diabetes management and glycemic control. This might be due to the development of problem-solving and planning skills, increased motivation for self-care, and better communication with healthcare providers [17]. A study by Ding et al. showed that better glycemic control was associated with better academic performance among children and adolescents with T1DM, and it suggested that better glycemic control may lead to improved cognitive function and increased school attendance, leading to better academic performance [18]. Our study found that children and adolescents with parents who had higher education levels scored significantly better in total QoL and treatment barriers, treatment adherence domains, and glycemic control than those who had parents with lower education levels. Parents with higher levels of education may have better knowledge and understanding of diabetes, which can influence their ability to manage their child's diabetes. Additionally, they may be more likely to engage in diabetes management behaviors that improve glycemic control, such as monitoring blood glucose levels, administering insulin, and following dietary recommendations. Furthermore, they may be more likely to utilize healthcare resources and seek diabetes management support for their child. Many studies in Egypt and Ethiopia support our results, particularly for mothers’ education levels. However, other studies in Greece found no association between maternal education level and QoL of children and adolescents [10,12]. Although children and adolescents of less-educated mothers had higher scores for total QoL and all subgroups except communication than those whose mothers had higher education levels, all results were not significant and may be attributable to mothers' occupational status.

Consistent with our results, a study conducted by Konstantaki et al. in Greece, which assessed the QoL of life of 87 children and adolescents with diabetes, showed that children on insulin pump therapy had higher QoL scores than those on MDI. However, there was no significant difference between the two groups [19]. Many studies have shown the superiority of insulin pumps over MDI in QoL and glycemic control. Al Shaikh et al. compared the QoL and glycemic control in 86 pediatric patients with T1DM who were either undergoing continuous subcutaneous insulin infusion (CSII) therapy or MDI. The study found that children on insulin pump therapy had significantly better QoL scores in diabetic symptoms and treatment control than those on MDI. Yet, the subjects on CSII were more worried than patients on MDI, and the two groups showed no significant difference in the scores for the communication domain and glycemic control [20]. In Kuwait, Mousa et al. [21] compared 236 children with T1DM on CSII and MDI to find significant improvement in glycemic control and QoL. Overall, evidence suggests that children and adolescents on insulin pump therapy have a better QoL than those on MDI. However, the effects of the type of therapy on glycemic control are unclear [22,23]. Therefore, the decision to use insulin pump therapy versus MDI should be based on individual patient needs and preferences and taken in consultation with a healthcare professional.

Insulin pump therapy can reduce the number of daily injections required for insulin delivery and may eliminate the need for frequent fingerstick blood glucose monitoring, thereby reducing the burden of diabetes management and improving the QoL. In addition, insulin pump therapy allows for more flexible insulin dosing and timing, which can lead to better glycemic control and fewer episodes of hypoglycemia. Insulin pumps deliver a continuous infusion of insulin, which is adjusted on the basis of the individual's insulin needs throughout the day. This allows for more precise insulin dosing and the ability to deliver insulin at different rates depending on the individual's activity level, food intake, and other factors. In contrast, MDI involves multiple daily injections of insulin, which may not provide sufficient flexibility in dosing and timing [24].

The AHA recommends that the HbA1c level for children should be maintained below 7.5% to prevent complications and hypoglycemia [25]. Approximately 17.13% of children and adolescents with T1DM in Alahsa achieved glycemic control with a mean HbA1c level of 9.04 ± 1.57. In Jeddah, Al Zahrani et al. stated that only 26.2% of 301 diabetic adolescents had HbA1c levels less than 8%, and the mean HbA1c level was 9.6 ± 1.9 [26]. The National Health and Nutrition Examination Survey (NHANES) reported that 17.1% of children and adolescents with diabetes in the United States had HbA1c levels of 9% or higher, indicating poor glycemic control [27]. The Hvidoere Study Group on Childhood Diabetes reported that 21% of European children and adolescents with T1DM had HbA1c levels > 9%, indicating poor glycemic control [28].

## Conclusions

Children and adolescents with T1DM in Alahsa had relatively good QoL. The findings also emphasized the importance of family-centered and psychosocial approaches for children and adolescents with T1DM. Females, young children, and patients with lower economic status may need attention depending on their need to improve their HRQoL. Conversely, adolescents may need more support to improve their glycemic control. Further adjustments for age-specific needs are essential to improve the HRQoL in this population. The cross-sectional nature of the study posed limitations in determining the temporality between QoL and glycemic control as well as the associated factors that affect the relationship between these factors.

In addition, interviews and survey data collection may have been associated with the risk of information bias. Additional observational research is recommended to address more psychosocial factors. Moreover, families and healthcare providers need to work together to ensure that children and adolescents with T1DM receive the best possible care and have access to the resources required to manage their condition. Frequent and routine assessments of QoL in this age group are crucial for better comprehensive diabetes care based on the recognition that diabetes is a complex and multifaceted condition that requires a holistic and individualized approach to management. By addressing all aspects of the disease, comprehensive care can help individuals with diabetes achieve optimal health outcomes and improve their QoL.
